# Buffalo Medical College

**Published:** 1852-09

**Authors:** 


*Buffalo Medical College.*—By the circular appended to this No. of the Journal, it will be perceived that the dissecting rooms in this College will be opened on the first Wednesday in November, for those students who desire to commence their winter labors at that time. This will give two months for prosecuting practical anatomy before the beginning of the regular term in January. Hospital instruction will also commence on the first Wednesday in November. We are authorized to state that anatomical material will be promptly supplied to any number of classes that may be formed after the dissecting rooms are opened.

Dr. Hugh B. Vandeventer, who has been appointed demonstrator of anatomy since the last session, will devote his entire attention to classes from the time of opening the dissecting rooms, through the preliminary and regular terms.

We are gratified to state that the portion of the college edifice originally designed for the museum, and hitherto unfinished, is now in progress of completion, and will be in readiness before the next session commences. When finished, it will be one of the most spacious and best arranged apartments for such an object in the country. It occupies one quarter of the entire building, is to be arched to the roof, surrounded by a gallery, and having space for a second gallery if required hereafter.

